# Physician experiences and barriers to addressing the social determinants of health in the Eastern Mediterranean Region: a qualitative research study

**DOI:** 10.1186/s12913-018-3408-z

**Published:** 2018-08-07

**Authors:** Labib Girgis, Gerald Van Gurp, David Zakus, Anne Andermann

**Affiliations:** 1 Department of Family Medicine, McGill University, Montreal, Canada; 2 Dalla Lana School of Public Health, University of Toronto, Toronto, Canada

**Keywords:** Social determinants of health, Social accountability, Health equity, Qualitative research, Eastern Mediterranean Region, Canada

## Abstract

**Background:**

While it is increasingly recognized that social determinants influence the health of patients and populations, little is known about how doctors in the Eastern Mediterranean Region can help their patients with these issues. Our study aimed to identify common social challenges faced by patients in Eastern Mediterranean countries, to assess what doctors are already doing to address these challenges, and to identify barriers and facilitators for addressing the social causes of poor health in Eastern Mediterranean countries with shedding some light on how does this compare to a developed country like Canada.

**Methods:**

We conducted a qualitative research study employing qualitative descriptive methodology. A purposeful sample as well as snowballing technique were used to recruit 18 physicians who were trained in Eastern Mediterranean countries but have since moved to Canada. Recruitment continued until data saturation was reached. A content analysis was carried out after transcribing the interviews.

**Results:**

The main social challenges identified in clinical care in Eastern Mediterranean Regions include poverty, illiteracy, domestic violence, and food insecurity. Doctors attempted to help their patients by providing free medical services and free medications, establishing a donation box, and referring to social workers and support services, where available. Cultural constraints, lack of time, and unavailability of referral resources were often cited as important barriers. Our participants stated that Canada is generally better in dealing with the social challenges than their countries of origin.

**Conclusions:**

Most study participants expressed their willingness to help patients in dealing with social challenges, and shared their experiences of tackling such issues, though there were also important barriers reported that would need to be overcome. Participants suggested that better addressing social challenges in clinical care would require educating both health care providers and patients about the importance of discussing the patient’s social environment as part of the health care encounter, as well as advocating for broader policy approaches by governments to address the underlying social problems.

## Background

There are many factors in addition to access to health care that can influence a person’s health and lead to a range of health problems encountered routinely by health care professionals. These factors are usually related to the patient’s conditions of daily living, for example, poverty, food insecurity, domestic violence, lack of education, and experience of discrimination. The World Health Organization (WHO) defines these social and economic circumstances affecting health as the social determinants of health (SDOH) [1].

Social accountability is increasingly becoming accepted as a primary responsibility of health care institutions, and doctors have an important contribution to make in tackling the social causes of poor health at multiple levels: 1) the patient level (micro), 2) the family and community level (meso), and 3) the broader societal and policy level (macro) [2–5].

While physicians in the Eastern Mediterranean Region (EMR) encounter patients every day who face plenty of social difficulties [6–16], little is known about whether and how doctors address such issues in their daily practice. In this particular context, what do doctors do when they attempt to help their patients in dealing with these underlying causes of poor health and what more could be done to better support marginalized and under-served patient groups?

The EMR as defined by the WHO [17] is a geopolitical group including the following nation states: Afghanistan, Bahrain, Djibouti, Egypt, Iran, Iraq, Jordan, Kuwait, Lebanon, Libya, Morocco, Palestine, Oman, Pakistan, Qatar, Saudi Arabia, Somalia, Sudan, Syria, Tunisia, United Arab Emirates, and Yemen. The vast majority of these (19/22 = 86.4%) are Arabic-speaking countries, with the exception of Afghanistan, Iran, and Pakistan. Geographically, most of these countries are located in the Middle East, with Djibouti, Egypt, Libya, Morocco, Somalia, Sudan, and Tunisia located in Northern Africa; and the rest of the countries are located in Western Asia and the Indian subcontinent (Pakistan).

This study aimed to explore the barriers and facilitators of addressing the social causes of poor health by doctors trained in EMR. In particular, to better understand what doctors are already doing in routine clinical practice to address the social challenges faced by their patients in these countries, and to recognize the main similarities and differences between doctors’ experiences in addressing SDOH in a clinical setting in EMR as compared to a Western context such as Quebec, Canada.

## Methods

### Study design

A qualitative descriptive research study methodology as described by Sandelowski and Neergaard was conducted within a naturalistic inquiry paradigm, using a series of semi-structured in-depth interviews [18, 19].

### Participant recruitment

A purposive maximum variation sampling technique was chosen to include a wide range of respondents by age, gender, time since graduation, country of origin, and duration in Canada [18, 20]. As well, we relied on a “snowball technique” to further identify potential respondents [20].

Only doctors trained in EMR who have since moved to Canada were included in this study, to permit an exploration of similarities and differences relating to addressing SDOH in these two different contexts. Study participants do not need to be working clinically in Canada, and may also draw upon their own experiences as a patient receiving clinical care in this new environment. Participant recruitment continued until data saturation was reached. In total, 18 interviews were conducted.

### Study setting

The study took place at the Department of Family Medicine at McGill University in Montreal, Canada. Ethics approval was obtained from the Institutional Review Board (IRB) of the St. Mary’s Research Centre, Montreal, QC, Canada. A written informed consent was obtained from each participant. The interviews were conducted between October and December 2016.

### Data collection

Eighteen in-depth interviews were carried out using a semi-structured interview guide consisting of open ended questions, as well as prompts, while allowing for unplanned questions to explore emerging issues raised during each interview. The 18 study interviews lasted about 30–60 min each, were conducted in English, and audio-recorded and then transcribed verbatim.

### Data analysis

Following transcription, the interviews were analyzed using qualitative content analysis as described by Graneheim and Lundman with a conventional approach as proposed by Hsieh and Shannon [21, 22]. Textual data from the interviews were sorted into five main content areas, which can be found in the results section, based on the use of a deductive coding frame as described by Crabtree and Miller [23]. Data coding was done by two independent persons. Codes and categories identified by the 2 independent researchers (LG & AA) were compared and any disagreements were resolved. The data analysis process was done manually without the use of analysis software. To ensure rigour, the four evaluating criteria (credibility, transferability, dependability, and confirmability) of research trustworthiness suggested by Lincoln and Guba have been followed [24].

## Results

A total of 18 doctors participated in the study. Details about participants can be found in Table 1.

**Table 1:** Description of participants (*n* = 18)

Gender		
12	66.7%	Female
6	33.3%	Male
Age		
0	0%	under 30 years
11	61.1%	30–39 years
4	22.2%	40–49 years
2	11.1%	50–59 years
1	5.6%	60 years and over
Medical specialty in country of origin		
9	50%	General practitioner
2	11.1%	Internal medicine
2	11.1%	Obstetrics & gynecology
2	11.1%	Urology
3	16.7%	One each for Anesthesia, Pediatrics, and Cardiovascular Surgery
Time since graduation		
10	55.6%	less than 10 years
7	38.9%	10–19 years
1	5.5%	20 years and more
Country of origin		
**7**	38.8%	Egypt
3	16.7%	Iran
3	16.7%	Syria
5	27.8%	One each from Pakistan, Lebanon, Tunisia, Palestine, Saudi Arabia
Time since moving to Canada		
0	0%	Less than 2 years
5	27.8%	2 years
3	16.7%	3 years
4	22.2%	4 years
5	27.8%	5 years or more
1	5.5%	Declined to mention
Occupation since moving to Canada		
5	27.8%	Researcher / Research Assistant
3	16.7%	Graduate Student (MSc or PHD)
4	22.2%	Physician (resident, restricted, or unrestricted practice)
2	11.1%	Full time preparing for medical equivalence exams
2	11.1%	Other occupation non-medical field
2	11.1%	Unemployed
Current place of residence in Quebec, Canada		
14	77.8%	Montreal and suburbs of Montreal
4	22.2%	Other cities in the Province of Quebec

### Main social challenges faced by patients in eastern Mediterranean countries

According to study participants, poverty is a common social challenge faced by patients in EMR. Often inter-related challenges faced by patients include lack of education, illiteracy, job precarity, food insecurity, stress, addictions, unstable families, domestic violence, and child abuse.

### Action on social determinants of health in clinical practice

While never having been formally taught how to do this in medical school, study participants shared many examples of ways in which they tried to help their patients in EMR with various social challenges, ranging from patient-level actions to broader community-level involvement.

#### Micro-level action at the patient level

Although not all study participants commonly enquired about the social challenges of their patients, those who did suggested that it was important to ask in an indirect way, and that a great deal of information could also be gathered from observing the patient’s general appearance and clothing. A 30-year-old female physician explained that: “You can tell if their hygiene is okay, if they are clean and well dressed, if it’s winter weather and the patient doesn’t wear a winter jacket.”

However, there are also times when it is important to confirm one’s intuitions by asking in a more direct way. A 50-year-old male physician suggests that: “Sometimes we cannot ask indirect questions, for instance, about domestic violence. In the end, if they don’t understand, then we have to ask directly.”

When social challenges were uncovered, it was not always straightforward knowing what to do. Few study participants had referred their patients to a social worker, mainly due to lack of availability of social workers where they practice, or because they felt that such referrals were generally ineffective. In this regard, a 35-year-old female physician said: “We have social workers but they do not do much, they work to pass their hours, but they are not trained.”

One thing that doctors could do is help patients to access care and needed treatments. Examples of such strategies included offering their medical services free-of-charge to patients in need, providing patients with medication samples or giving away medication for free, and even paying for a patient’s other treatment costs if they were unable to cover the costs themselves. A 44-year-old general practitioner justifies paying the prescription costs for her patients in need as follows: “I did not have any idea how I can help them. Sometimes I can pay the prescription.”

#### Meso-level action at the institutional level

Beyond action at the doctor-patient level, participants also talked about charitable efforts by institutions which were important strategies used to support patients. Some medical institutions established a donation box funded by doctors to help patients in need. Study participants also mentioned that the wealthy in their community support poor people by offering financial aid. A 50-year-old female physician stated: “We have in the charity hospital rich people who pay for the poor people.”

#### Macro-level action at the community and societal level

Most governments of EMR offer governmental health insurance. However, in many cases the coverage is restricted to certain populations and covers only a small number of procedures. Thus, religious organizations, such as mosques and churches played an important role in supporting underserved populations. According to a 38-year-old physician: “There are some community associations that help people who have chronic illnesses, the church also, there are many community associations related to the church, they even give emotional support to people in bad situations.”

In addition to helping patients secure financial assistance, there were also study participants who provided their own personal time and energy to support their community and engage in community-based initiatives such as health care campaigns in remote areas and educational sessions for underserved regions. This was stated by one of the participants as follows: “In [name of participant’s country] … some health workers go to remote areas to help people with very low economic levels who have zero access to medical services… doing that for free to help the community… we educate them how to prevent some diseases.”

### Barriers addressing social determinants in Eastern Mediterranean countries

While there were examples of how doctors can help to address the social causes of poor health at multiple levels, there were also many deep-rooted barriers and concerns raised.

#### Cultural constraints and social norms

Study participants considered that some doctors may be unwilling to ask about the social challenges of their patients because they do not want to breach existing cultural norms. For instance, when doctors ask about private matters, it could be mistakenly perceived as “sticking one’s nose” in the patient’s personal life. A 50-year-old male physician explained that he generally does not ask about or intervene if he suspects a case of domestic violence since there is the perception of the social norm that: “Domestic violence in our country is accepted. This is the problem. [..] it is everywhere. You don’t agree with it, but everyone around you agrees with it, so you will be the exception [..] If you try to criticize you will be attacked.”

### Limited access to universal primary health care and referral resources

Another important barrier to taking action on the social causes of poor health relates to the pressures on existing physicians to see large numbers of patients resulting in relatively small amounts of time with each patient due to few family doctors available to provide primary care. As well, there is also a lack of effective social support organizations that can be used as referral resources for patients who are identified as having social challenges. A 37-year-old male physician explained that, although doctors may want to refer to social support organizations “Unfortunately, there are none. The role of community associations in supporting vulnerable patients is so minimal. They lack legal reinforcement; they don’t have any legal support which means they are not effective. They don’t have the power or legislation to support the vulnerable patients.”

Even if there are some organizations that may exist, another barrier is that doctors are often unfamiliar with the services available in their area.

### Governmental policies and structural factors

Ultimately, the social challenges that patients face are mainly due to limited or non-equitable distribution of national resources, and absent or restricted social security nets. These are often considered to be factors beyond the doctor’s control, and therefore study participants often feel “helpless” to influence changes at this level. This feeling was expressed by one of the male physicians as follow: “After a while, [doctors] become exhausted, they don’t intervene, they think that it is dangerous for them, maybe it is not their job, they risk their time, they don’t receive anything… so why should they spend this value of time on things that they cannot do anything for it”.

### Similarities and differences between Canada and Eastern Mediterranean countries

Canada is recognized by study participants as a country where doctors are better equipped to support patients in dealing with their social challenges. When the participants were asked to explain why this may be, they mentioned a number of facilitating factors. These include, more social support resources being available in Canada, that doctors are more likely to be trained to ask about the SDOH during the clinical encounter, that doctors are often more familiar with locally-available social support organizations, and that there are also more social workers available in health care institutions to support patients, particularly in dealing with the most challenging situations. A 50-year-old female participant who is currently a family medicine resident in Quebec says: “We know some organizations. For the pregnant women, we have here in the region a lot of organizations for nutrition, for further types of support, and they give us a lot of addresses for that … for the organizations … We have a list of organizations.”

Nevertheless, in spite of these facilitators, some barriers were also identified in the Canadian setting. The most important barrier is cultural diversity which might become an obstacle to asking about and addressing SDOH when caring for patients from different cultural backgrounds. Thus, the patient might feel more comfortable to discuss his/her social issues with a provider from the same cultural background, and vice versa. This was implied by the same family medicine resident when she said: “You have to know this culture and how to deal with it. I know about Arabic culture. I know how to deal with these people. But, I do not know a lot about the Chinese culture for example. We have a lot of Chinese patients. So, we have to know these cultures.”

### Structural changes needed to support action on social determinants

Participants suggested that a number of reforms and changes are needed for doctors in EMR to be better equipped to support their patients in overcoming the underlying social causes of poor health.

Educating both health workers and patients on the value of discussing and addressing social challenges during the clinical encounter would be an important first step. This could be done by integrating education about SDOH into medical school curricula, training physicians to ask about SDOH, applying guidelines or protocols to assist in dealing with social challenges, and familiarizing doctors about referral resources and particularly about local social support organizations that are available. For instance, a female participant said: “I think in the medical schools, they should receive training or sessions about social determinants. And they should know what are the social determinants, they should know where to refer and by the time they graduate, they will have a list of resources and referral options.”

As well, changing patients’ perceptions and raising their awareness about the value of discussing their social problems with their health care providers would be important. A 38-year-old female participants explained: “We have to talk to our patients more, we have to give a little time to our patients to change their mind, to tell them that it’s our role as physicians to know these things because it has an impact on their health issues”.

Participants also emphasized the importance of providing suitable financial support as well as raising the doctors’ salaries. A 32-year-old male participant emphasized this when he said: “Better education and training programs need financial support. The education of physicians to face social challenges needs money.”

Study participants suggested that supporting patients requires strengthening the doctor-patient relationship and improving communication skills. Doctors should listen carefully and avoid criticizing or judging their patients. They have to put themselves in the patient’s “shoes” when asking about the SDOH, and be sensitive to local contextual considerations. However, participants felt that they alone could not do everything, and that much larger societal reforms are also needed. For instance, changes at the governmental level such as copying the Canadian social assistance system, greater collaboration between government and non-governmental organizations (NGOs), and applying pressure on the government to address the underlying social challenges that patients face. A 31-year-old male participant said: “As a physician, we have to apply pressure on the government who is not willing to finance these projects that are important for the people.”

## Discussion

While participants considered poverty as a predominant social challenge in their countries, there is very little written in the medical literature of the EMR about managing patients living in poverty and suffering from poverty-related challenges. The literature that does address social challenges in this region mostly deals with domestic violence [7, 10–13, 15, 16, 25], child abuse [6, 8, 9] and addictions [14]. While these are also very important issues, there is clearly a knowledge gap in the literature that needs to be addressed by future research.

Study participants generally expressed their willingness to help their patients in dealing with their social challenges, and even used novel approaches that are not described in industrialized countries like Canada [26]. These approaches include providing free medical care, paying the cost of medications, and establishing a donation box in clinics and hospitals. Thus, study participants often attempted to assist their patients using their own limited financial resources due to the unavailability of other helpful and effective measures.

Moreover, there are examples of how participants attempted to tackle the social challenges of their patients at the 3 levels described by the social accountability framework [2–5]. This included actions at the patient level, at the family and community level, as well as at the broader societal and policy level. Although the participants have not received formal education or training about social accountability concepts and its application to the healthcare field, they intuitively had developed strategies that could be broadly categorized into the different levels of this framework, though of course there is also much more that they could do and that is being done in other contexts [27].

Barriers in terms of addressing the SDOH in clinical practice in the Eastern Mediterranean context are mostly consistent with those found in the literature. However, there was greater emphasis on cultural constraints and social norms, and less recognition that formal education or training to deal with the social challenges of patients in clinical practice can promote change [8, 9, 11, 15], even if the barriers to act upon the SDOH are often systemic and also need to be addressed at the highest levels of government.

Indeed, study participants proposed several structural changes that could be applied in their countries of origin to improve action on the SDOH. In particular, participants expressed the need to incorporate teaching about SDOH into medical school curricula, which is consistent with the scientific literature in EMR [28] and in industrialized countries such as Canada [29–32].

### Study limitations

This study is an exploratory qualitative study. It is not a representative sample of all 22 EMR countries, which are characterized by cultural, linguistic, geographical, economical, and political diversity. Yet, we did obtain a diverse sample with a wide range of views, and though generalizability was not our target; we were able to explore the experiences and perceptions of our participants, which sheds light on this previously understudied area of inquiry.

The period of time the study participants remained away from their countries of origin after moving to Canada might negatively affect their answers as they may be out of touch with recent developments in the region. However, we believe this limitation does not influence the validity of participant responses as 12 out of 18 participants (66.7%) moved to Canada within the last 4 years.

The study participants may also have some degree of negative bias against their countries of origin that led to their migration, though physicians moving to Canada are generally economic migrants rather than refugees. Indeed, many participants shared positive experiences in supporting their patients before moving to Canada. These experiences can serve as a form of South-North learning to help Canadian health workers identify and act upon social challenges of the diverse patient populations that they serve. This can also promote greater cultural competence to help health workers address marginalization and inequity in the Canadian context.

## Conclusion

Better addressing social challenges in clinical care would require educating both doctors and patients about the importance of discussing the patient’s social environment as part of the health care encounter, as well as advocating for broader policy approaches by governments to address the underlying social causes of poor health. The Canadian social support system and the way in which medical education and training is increasingly focused on social accountability can be used as inspiration for promoting action on health equity worldwide, while recognizing that there are already tremendous efforts and innovations that exist within low- and middle-income country contexts, as evidenced by the EMR experience, that can be further developed in future.

## Abbreviations

EMR: Eastern Mediterranean Region
NGOs: Non-governmental organizations
SDOH: Social Determinants of Health
WHO: World Health Organization
